# A panel of DNA methylated markers predicts metastasis of pN_0_M_0_ gastric carcinoma: a prospective cohort study

**DOI:** 10.1038/s41416-019-0552-0

**Published:** 2019-08-21

**Authors:** Zhaojun Liu, Xiaojing Cheng, Lianhai Zhang, Jing Zhou, Dajun Deng, Jiafu Ji

**Affiliations:** 1 Key Laboratory of Carcinogenesis and Translational Research (Ministry of Education/Beijing), Division of Etiology, Peking University Cancer Hospital and Institute, Fu-Cheng-Lu, No. 52, Haidian District, Beijing, 100142 China; 2 Department of Gastrointestinal Surgery, Peking University Cancer Hospital and Institute, Fu-Cheng-Lu, No. 52, Haidian District, Beijing, 100142 China

**Keywords:** Tumour biomarkers, Predictive markers

## Abstract

**Background:**

The aim of this prospective study was to evaluate the feasibility of predicting GC metastasis using *CDH1*, *GFRA1*, *P16* and *ZNF382* DNA methylation as biomarkers.

**Methods:**

198 GC patients without metastasis at the time of surgery resection were recruited into the double-blind cohort (NCT02159339). Gene methylation was analysed using MethyLight assays. GC metastasis and survival data were obtained from 178 patients with 94.7% compliance during follow-up.

**Results:**

Twenty six cases of metastasis and 5 cases of recurrence were observed in 178 cases (17.4%) during the follow-up (median, 62.7 months). The GC metastasis rate for *GFRA1* methylation-positive patients was significantly reduced compared with *GFRA1* methylation-negative patients (odds ratio [OR]: 0.23, 95% confidence interval [CI] 0.08–0.66). Similar results were also observed using *ZNF382* methylation as a predictor (OR: 0.17, 95% CI 0.06–0.47). A risk score including methylation of *GFRA1* and *ZNF382* was generated. The metastasis rate was significantly increased in high-risk GC patients (OR: 4.71, 95% CI: 1.85–12.00). GC patients with high risk had a shorter overall survival, especially for patients with stage I GC (*P* = 0.024).

**Conclusions:**

The combination of *GFRA1* and *ZNF382* methylation is a biomarker panel for the prediction of GC metastasis.

## Background

Gastric carcinoma (GC) is the third leading cause of cancer-related death worldwide, with 723,000 deaths reported in 2012.^1^ Approximately 30–40% of patients with GC undergo relapse, including local recurrence or distant metastases, after surgery.^2^ The prognosis of metastatic GC is poor, with a 5-year overall survival (OS) rate of ~4% and a median survival of ~9–10 months,^3^ thus underscoring the need for early, reliable predictive biomarkers.

Cancer metastasis can be classified into two phases: a clinically latent stage and a manifest stage. The period of clinically undetectable minimal residual disease offers a time window to prevent metastasis.^4,5^ Thus, it is important to detect the lesion at the latent stage. Although comprehensive studies have identified several genes associated with GC development and progression, a sensitive and specific biomarker capable of predicting prognosis and likelihood of metastasis is lacking.^6–9^

DNA methylation is one of the most common epigenetic alterations, and aberrant methylation can be an early event in cancer progression, indicating its potential as a biomarker for cancer detection.^10^ In our previous study, we demonstrated that the *GFRA1* and *ZNF382* genes were methylated in human GC tissues and significantly associated with a low risk of GC metastasis in Chinese, Japanese, and Korean patients.^11^ *P16* (CDKN2A) is one of the most frequently deleted genes in cancer genomes.^12^ Recently, genetic and epigenetic inactivation of *P16* has been proven to be a driver for cancer metastasis.^13,14^ The *CDH1* gene participates in the establishment and maintenance of intercellular adhesion, cell polarity, and tissue architecture. The loss of *CDH1* expression promotes cancer metastasis through the epithelial–mesenchymal transition (EMT) mechanism. Decreased *CDH1* expression through promoter DNA methylation is frequently detected in many cancers and is associated with poor prognosis.^15,16^ To evaluate the feasibility of predicting GC relapse using DNA methylation changes within CpG islands at these gene promoters, in the present study, the association between gene methylation and GC metastasis/recurrence was prospectively studied among 198 GC patients without metastasis at the pN_0_M_0_ stage at the time of surgical resection.

## Methods

### Patients and study design

**Fig. 1:**
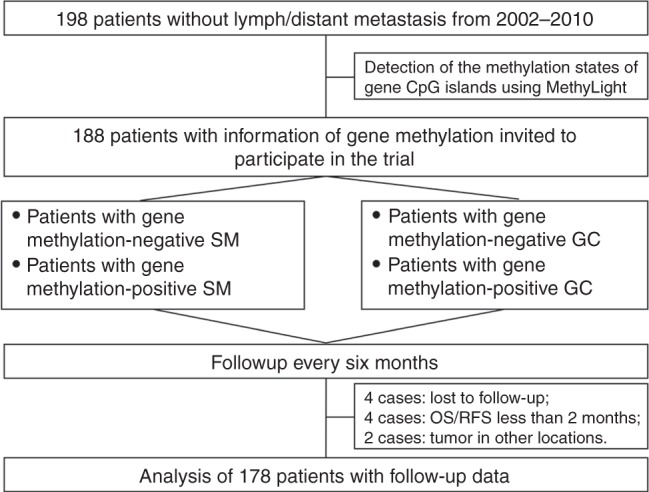
Participant flow diagram

In total, 198 pN_0_M_0_ GC patients who underwent surgical treatment at Peking University Cancer Hospital & Institute between 2002 and 2012 were enrolled in the cohort. The 2010 UICC-tumour-node-metastasis (TNM) system was used for the classification of GCs.^17^ All GC cases were recruited into the study based on the following inclusion criteria: histological diagnosis of gastric adenocarcinoma; no lymph node and distal metastasis at surgical resection time; availability of frozen, fresh GC and corresponding surgical margin (SM) samples; and no neoadjuvant chemotherapy. All patients underwent radical resection surgery. Adjuvant chemotherapies were recommended for patients with stage III and high-risk stage II disease after surgical resection. The chemotherapy regimens were primarily fluorouracil-based, with or without leucovorin, paclitaxel or oxaliplatin. The Institutional Review Board of the Peking University Cancer Hospital and Institute approved this study (ethics approval document #2006021). All patients provided written informed consent (Fig. 1). The study is registered in the U.S. National Institutes of Health Clinical Trials Protocol Registration System in accordance with the criteria outlined by the International Committee of Medical Journal Editors (trial number NCT02159339, available at http://ClinicalTrials.gov).

### DNA methylation data in GC tissues from TCGA dataset

The DNA methylation data (by Infinium HumanMethylation450) for 376 patients with GC and related clinical information were downloaded from the Cancer Genome Atlas (TCGA) database.^18^ The methylation level for each CpG site was expressed as β value, calculated as M/(M+U), where M is the signal from methylated beads, and U is the signal from unmethylated beads at the targeted CpG site. When the β value for a CpG site is >0.6, it is classified as methylation-positive CpG (mCpG). The total number of mCpG sites, 31 and 11 CpGs/probes for *GFRA1* and *ZNF382* CpG islands in the Ilumina HumanMethylation450 array, was used to represent the *GFRA1* or *ZNF382* methylation level for each sample, respectively. A median dmCpG number of 1 was used as the cut-off value to define *GFRA1*m and *ZNF382*m. A sample containing ≥1 mCpG site(s) was classified as *GFRA1m*-positive (or ZNF382m-positive) sample. Only the data for patients with survival information were used to analyse the correlation between the methylation status of target CpG islands and GC metastasis.

### Patient follow-up

GC metastasis, including local recurrence, and patient survival states were assessed every 6 months. GC metastasis/recurrence was radiologically diagnosed via abdominal computed tomography (CT) and then confirmed pathologically in surgical or excisional biopsy specimens or clinically in follow-up abdominal CT. The primary endpoint was relapse-free survival (RFS), defined as the time from the date of surgery to the date of metastasis. The secondary endpoint was overall survival (OS), defined as the time from the date of surgery to the date of death from any cause or censored at the last follow-up.

### DNA extraction and bisulphite conversion of genomic DNA

Genomic DNA was extracted using the QIAamp DNA Mini Kit (Qiagen, Cat# 51306, Hiden, German). Bisulphite conversion was performed by adding 5 M sodium bisulphite to 1.8 μg DNA samples.^19^ During the bisulphite modification process, genomic DNA from RKO cells containing methylated *CDH1, GFRA1, P16*, and *ZNF382* genes was used as the methylation-positive control, whereas genomic DNA from GES-1 cells containing unmethylated *CDH1, GFRA1, P16*, and *ZNF382* genes was used as the methylation-negative control.

### Quantification of gene methylation using the MethyLight assay

The methylation states of *CDH1, GFRA1, P16*, and *ZNF382* CpG islands were determined using MethyLight assays.^11,20,21^ Gene-specific probes labelled with 6FAM and TAMRA were used to quantify the relative copy number of methylated alleles compared with the *COL2A1* control.^22^ Briefly, oligonucleotide primers were used to specifically amplify bisulphite-converted DNA, and methylated gene-specific probes were used to detect the amplicon number during extension (Supplementary Table 1).^11,20,21^ The exact locations of the analysed CpG sites within these genes are illustrated in Supplementary Fig. 1. The PCR product number of a target gene is proportional to the number of methylated templates. For the internal reference gene *COL2A1*, gene-specific CpG-free primers and probes were used to amplify *COL2A1* alleles independent of its methylation status. The sequences of the primer set, and gene-specific probes can be found in Supplementary Supplementary Table 1. To ensure accurate PCR results, sufficient amounts of input DNA template are required, and only samples with the reference *COL2A1* Ct value < 29.3 were considered as informative in gene methylation, as previously reported.^23^ PCR was performed individually for each gene, including the *COL2A1* reference, in duplicate. The reproducibility of the MethyLight PCR was regularly monitored according to amplification curves (Supplementary Fig. 2).

### Statistical analysis

The chi-square test and forward binary logistic regression analysis were used to analyse the association between gene methylation and the clinicopathological features for univariate and multivariate analyses. GC metastasis-predicting performance was assessed using receiver operating characteristic (ROC) curve analysis. When the proportion of methylated alleles was> 6.84% for *ZNF382* and >8.64% for *GFRA1*, they were defined as *ZNF382* and *GFRA1* methylation-high (ZNF382m-high and GFRA1m-high). Otherwise, they were defined as methylation-low (ZNF382m-low and GFRA1m-low). The log-rank test was used to compare RFS and OS times between groups. Cox proportional hazards models were used to identify independent predictors of survival (month) with adjustment for relevant clinical covariates. Relevant predictor variables for the Cox regression model were identified by using a stepwise selection procedure from the Akaike information criterion (AIC).^24^ Comparisons between different prognostic models were evaluated by concordance index (C-index). All statistical tests were two-sided, and *P* < 0.05 was considered statistically significant. Analyses were performed in R 3.4.3 (R Core Team 2017) using the packages compareC, rms, survival and SPSS (version 16.0).

## Results

### Patients' basic information

**Fig. 2:**
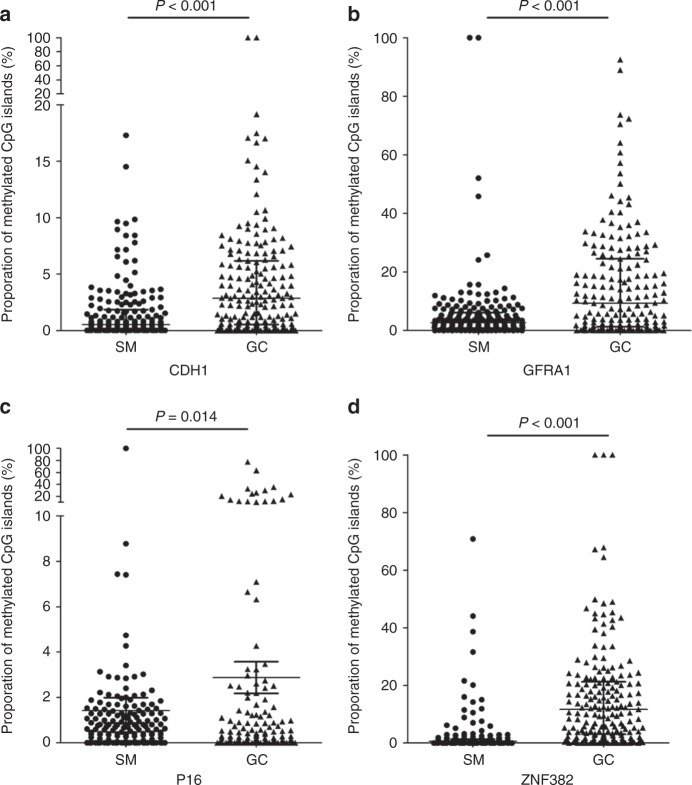
Comparison of the proportions of methylated*-*gene alleles in gastric carcinoma (GC) and corresponding surgical margin (SM) samples from 151 GC patients using MethyLight analysis. **a** CDH1; **b** GFRA1; **c** P16; **d** ZNF382

Information on the methylation status of target CpG islands was successfully obtained from 188 of 198 patients in MethyLight analyses. Ten of 188 patients were excluded from the final dataset. These 10 cases included 4 cases who died with less than two months of follow-up due to surgical complications, 2 cases with second tumours in other locations, and 4 cases lost due to changes in contact information during follow-up (ranging from 2.1 to 138.3 months, with a median 62.7 months). The follow-up compliance was 94.7%. GC relapse was observed in 31 cases (17.4%; including 26 cases with metastasis and 5 cases with local recurrence) during the follow-up (median for postsurgical metastasis, 62.6 months). The relapse rate was significantly increased in patients with stage T3 and T4 GC (*n* = 127) compared with patients with stage T1 and T2 GC (*n* = 51) [21.3% versus 7.8%, *P* = 0.047]. The methylation-positive rates for the *CDH1*, *GFRA1*, *P16*, and *ZNF382* genes were 87.1%, 93.8%, 68.0%, 92.7% in SMs, respectively, and 79.2%, 94.4%, 51.1%, 97.2% in GCs, respectively. The average proportion of methylated *CDH1*, *GFRA1*, *P16*, and *ZNF382* alleles was strikingly increased in GC samples compared with SM samples (*Ps* < 0.05) (Fig. 2).

### Association between gene methylation and metastasis of pN_0_M_0_ GCs

**Fig. 3:**
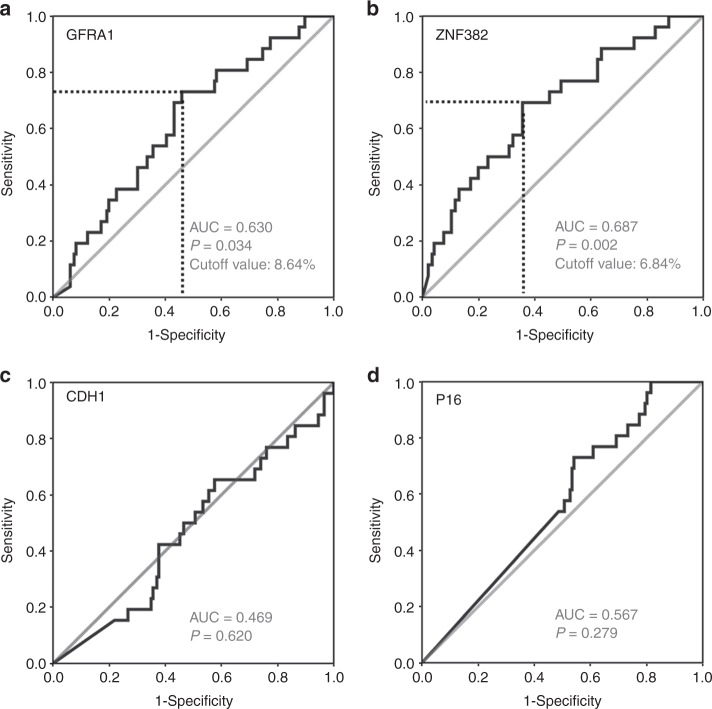
ROC curves for the prediction of GC metastasis by GFRA1, ZNF382, CDH1 and P16 methylation positivity as biomarkers. The cut-off value to define methylation-positive patients is noted. **a** GFRA1; **b** ZNF382; **c** CDH1; **d** P16

Because of the different biological mechanism between metastasis and local recurrence, we excluded the 5 recurrence cases from the 178 GC patients in the metastasis analysis. To evaluate the performance of gene methylation in predicting GC metastasis, ROC curves were initially calculated using information data for *CDH1*, *GFRA1*, *P16*, and *ZNF382* methylation in GC samples. The areas under the ROC curves were 63.0% for GFRA1m (*P* = 0.034) and 68.7% for ZNF382m (*P* = 0.002) (Fig. 3a, b). No metastasis difference was found between GC patients with and without *CDH1* or *P16* methylation in the GC samples (Fig. 3c, d). Thus, according to the ROC curves, >6.84% and >8.64% of the proportion of methylated alleles were selected as the cut-off values to define ZNF382m-high and GFRA1m-high, respectively. The analysis results showed that the GC metastasis rate for patients with ZNF382m-high GC was significantly lower than for patients with ZNF382m-low GC in univariate analysis (9.7% versus 22.9%, odds ratio [OR]: 0.25, 95% confidence interval [CI] 0.10–0.60; *P* = 0.002). The difference remained significant in multivariate analysis after adjustment for age, gender, location, differentiation, vessel embolus, stage, and history of adjuvant chemotherapy in multivariate analysis (adjusted OR: 0.17, 95% CI 0.06–0.47; Supplementary Table 2). Similarly, the difference in metastasis between patients with GFRA1m-high GC and patients with GFRA1m-low GC was also statistically significant in univariate analysis (8.1% versus 21.8%, OR: 0.31, 95% CI 0.12–0.79; *P* = 0.014) and multivariate analysis (adjusted OR: 0.23, 95% CI 0.08–0.66; Supplementary Table 3). Methylation of 4 genes from 173 GCs was selected for the statistical model. To investigate whether a combination of these methylation markers has a synergistic effect on predicting GC metastasis, we constructed a prognostic risk grading model based on the methylation status of 4 genes. The best risk score modelled on these data encompassed the methylation of *GFRA1* and *ZNF382*. The c-index of the model was 0.687. According to the data described above, both ZNF382m-low and GFRA1m-low were defined as metastasis risk factors. A subject with no risk factor was subclassified into the low metastasis risk group; a subject containing 1 risk factor was subclassified into the moderate-risk group, and a subject containing 2 risk factors was subclassified into the high-risk group. A total of 76 (43.9%), 37 (21.4%), and 60 (34.7%) subjects were subclassified into the low, moderate, and high-risk groups, respectively. As expected, the ratio of patients with GC metastasis was gradually and significantly increased along with increased risk grades (6.6%, 13.5%, and 26.7%; linear-trend test, *P* = 0.001). The sensitivity and specificity of both ZNF382m-low and GFRA1m-low for the prediction of GC metastasis were 61.5% and 70.1%, respectively. The risk grade remained as an independent predictive factor, even after adjusting for age, gender, location, differentiation, vessel embolus, TNM stage, and history of adjuvant chemotherapy in the multivariate analysis (adjusted OR: 4.71, 95% CI: 1.85–12.00; Table 1).

**Table 1:** Prevalence of gastric carcinoma (GC) metastasis during follow-up in patients in different risk groups

	GC metastasis rate (%)		OR (95% CI), high vs. low & moderate risk groups	
	Patients in the low & moderate risk groups	Patients in the high risk group	Univariate analysis	Multivariate analysis
Sex				
Male	7/75 (9.3)	13/46 (28.3)	**3.83 (1.40–10.49)**	**5.38 (1.74–16.68)**
Female	3/38 (7.9)	3/14 (21.4)	3.18 (0.56–18.09)	
Age (yrs)				
<60	4/49 (8.2)	13/34 (38.2)	**6.96 (2.03–23.94)**	**9.81 (2.50–38.46)**
≥60	6/64 (9.4)	3/26 (11.5)	1.26 (0.29–5.47)	
Location				
Non-cardiac	6/82 (7.3)	8/33 (24.2)	**4.05 (1.28–12.81)**	**5.08 (1.51–17.06)**
Cardiac	4/31 (12.9)	8/27 (29.6)	2.84 (0.75–10.81)	
Different.				
Well/ Mod.	3/42 (7.1)	7/20 (35.0)	**7.00 (1.58–31.09)**	
Poor	7/69 (10.1)	8/36 (22.2)	2.53 (0.84–7.67)	
Vessel embolus				
Negative	7/94 (7.4)	12/49 (24.5)	**4.03 (1.47–11.05)**	**4.13 (1.50–11.40)**
Positive	2/18 (11.1)	3/9 (33.3)	6.40 (0.89–45.99)	
TNM Stage				
I	1/34 (2.9)	3/17 (17.6)	7.07 (0.68–73.99)	
II	8/65 (12.3)	11/35 (31.4)	**3.27 (1.17–9.13)**	**4.71 (1.55–14.38)**
III	1/14 (7.1)	2/8 (25.0)	4.33 (0.33–57.65)	
Chemotherapy				
No	3/64 (4.7)	7/33 (21.2)	**5.47 (1.31–22.84)**	**5.22 (1.20–22.62)**
Yes	6/46 (13.0)	9/27 (33.3)	**3.78 (1.09–13.14)**	**4.80 (1.30–17.78)**
(Total)	10/113 (8.8)	16/60 (26.7)	**3.75 (1.58–8.90)**	**4.71 (1.85–12.00)**

Bold values indicate statistical significance *p* < 0.05

The ROC curves were calculated using methylation data of *CDH1*, *GFRA1*, *P16*, and *ZNF382* to further evaluate the performance of these gene methylation to predict local GC recurrence in 152 GC patients. The areas under the ROC curves were 85.7% for GFRA1m (*P* = 0.007) and 77.3% for CDH1m (*P* = 0.038) in SMs.

### Validation of predictive effect of the target biomarkers

The predictive value of selected methylation biomarkers for GC metastasis was further confirmed using internal cross validation. Patients were divided into two parts according to the date of surgical operation. Patients who underwent operation before and after 2008 were classified as testing-1 and testing-2 cohort, respectively. In testing-1 cohort, the analysis results showed that the GC metastasis rate for patients with ZNF382m-high GC was significantly lower than for patients with ZNF382m-low GC in univariate analysis (5.5% versus 29.0%, adjusted OR: 0.07, 95% CI: 0.01–0.45). The GC metastasis rate for patients with GFRA1m-high GC was much lower than for patients with GFRA1m-low GC (9.1% versus 29.0%), but not significant. Similar result was acquired in testing-2 cohort. The GC metastasis rate for patients with ZNF382m-high GC was much lower than for patients with ZNF382m-low GC (10.4% versus 23.1%), but not significant. The difference in metastasis between patients with GFRA1m-high GC and patients with GFRA1m-low GC was statistically significant in univariate analysis (7.1% versus 24.4%, adjusted OR: 0.17, 95% CI: 0.03–0.91). As expected, the ratio of patients with GC metastasis was significantly increased in the high-risk group than those in the low- and moderate-risk groups, both in testing-1 cohort (8.2% versus 28.0%, adjusted OR: 5.48, 95% CI: 1.19–25.31) and testing-2 cohort (9.6% versus 25.7%, adjusted OR: 3.49, 95% CI: 0.88–13.91) (Supplementary Table 4).

To further validate the predictive effect of the selected biomarkers, we analysed the methylation array data for 376 GC patients in TCGA database (Supplementary Table 5). The analysis results showed that the distant metastasis rate for patients with ZNF382m-positive GC was significantly lower than that for patients with ZNF382m-negative GC (3.9% versus 8.8%, OR: 0.42, 95% CI: 0.17–1.00; *P* = 0.047). Similarly, the distant metastasis rate for patients with GFRA1m-positive GC was also lower, albeit non-significantly, than that for patients with *GFRA1* unmethylated GC (4.3% versus 8.8%; *P* = 0.067).

In addition, ZNF382m- and GFRA1m-negative statuses were also defined as risk factors for metastasis and used to sub-classify the 376 TCGA patients into three groups as described above: 172 (45.7%), 78 (20.7%), and 126 (33.5%) subjects in the low-, moderate-, and high-metastasis risk groups. As expected, the percentage of metastatic GC patients was significantly higher in the moderate- and high-risk groups than that in the low risk group (10.3% vs. 4.0%; *P* = 0.016). The risk grade remained an independent predictive factor, even after adjusting for age, gender, race, location and invasion depth in the multivariate analysis (adjusted OR: 3.25, 95% CI: 1.02–10.31).

### Survival analysis for GC patients with different methylation status of target genes

**Fig. 4:**
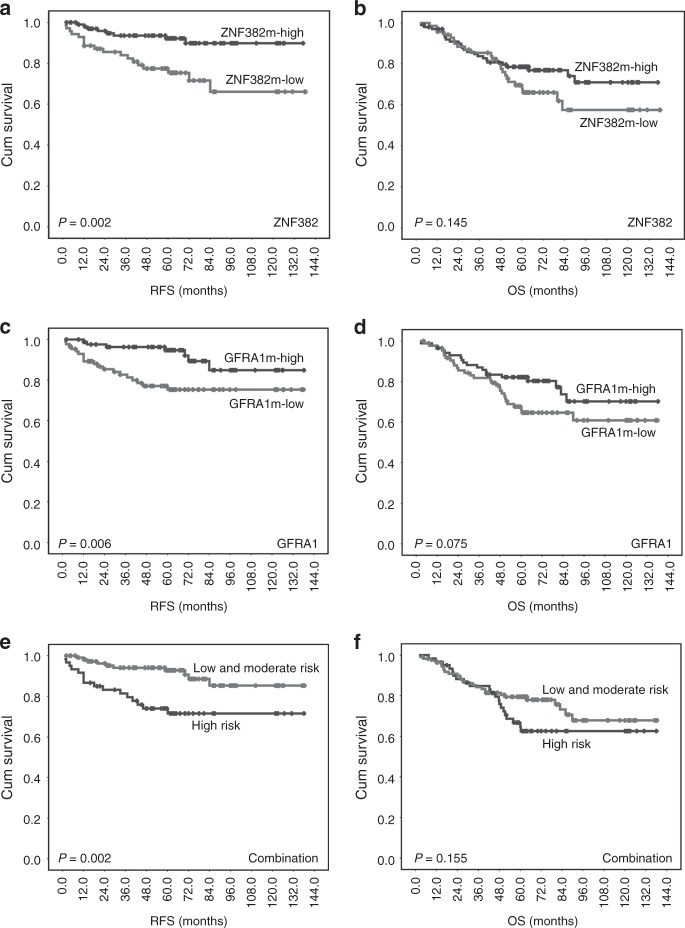
Kaplan–Meier survival curves of gastric carcinoma (GC) patients with different GFRA1/ZNF382 methylation states and numbers of risk factors in GC tissues. **a**, **c**, **e** Relapse-free survival (RFS); (**b, d, f**) Overall survival (OS)

To investigate the survival difference between the metastasis onset time for GC patients with different gene methylation statuses during the follow-up, RFS was analysed using Kaplan–Meier analysis. The results showed that patients with ZNF382m-high GC had a significantly longer RFS than those with ZNF382m-low GC in univariate analysis (hazard ratio [HR]: 0.28, 95% CI 0.12–0.66) and multivariate analysis (adjusted HR: 0.24, 95% CI 0.09–0.61) (Fig. 4a; Supplementary Table 6). Similarly, the RFS of patients with GFRA1m-high GC was also significantly longer compared with those with GFRA1m-low GC (adjusted HR: 0.26, 95% CI 0.10–0.72) (Fig. 4c). The OS of patients with ZNF382m-high or GFRA1m-high GC was longer than those with ZNF382m-low or GFRA1m-low GC, although the difference was not significant (Fig. 4b, d). Stratification analysis showed that patients with GFRA1m-high had a significantly longer OS for patients with stage I-GC (*P* = 0.022, the log-rank test), although this difference was not significant in multivariate analysis. No statistically significant association was detected between RFS or OS and methylation of the remaining genes.

Significant prognostic stratification for RFS was provided by the risk grading based on the methylation status of *GFRA1* and *ZNF382*. Patients in the high-risk group had a shorter RFS than those in the low- and moderate-risk groups (*P* = 0.002, log-rank test) (Fig. 4e). The risk grade was a significant independent prognostic factor for RFS of these pN_0_M_0_ GC patients, even after adjusting for age, gender, location, differentiation, vessel embolus, TNM stage, and history of adjuvant chemotherapy in the multivariate Cox proportional hazard analysis (HR: 3.38, 95% CI: 1.39–8.19) (Table 2). While GC location and pTNM stages were significantly associated with patients' OS, patients in the high metastasis risk group tended to have a shorter OS than patients in the low- and moderate-risk groups (Fig. 4f), but this difference was not statistically significant (Table 2). Stratification analysis showed that GC patients with high risk had a shorter OS, especially for patients with stage I GC (log-rank test, *P* = 0.024). The same trend was observed in multivariate analysis (adjusted HR: 8.30, 95% CI: 0.93–74.42, *P* = 0.059), although it was not significant. In the internal cross-validation analyses, similar survival difference was also observed among these GC patients in the testing-1 and testing-2 sets (Supplementary Fig. 3). Such survival difference was also observed between patients with ZNF382m-positive or GFRA1m-positive GC and patients with ZNF382m-negative or GFRA1m-negative GC from TCGA databases (Supplementary Fig. 4).

**Table 2:** Univariable and multivariable Cox regression analyses of methylation signatures for relapse-free survival (RFS) and overall survival (OS) of gastric carcinoma patients with different metastasis risk grades and clinicopathological characteristics

Clinical parameter	Relapse-free survival (RFS)				Overall survival (OS)			
	Univariate analysis		Multivariate analysis		Univariate analysis		Multivariate analysis	
	HR (95% CI)	*P-*value	HR (95% CI)	*P-*value	HR (95% CI)	*P-*value	HR (95% CI)	*P-*value
Metastasis risk grade (high- vs. low- & moderate-risk)	**3.27 (1.48–7.21)**	**0.003**	**3.38 (1.39–8.19)**	**0.007**	1.51 (0.85–2.67)	0.158	1.410 (0.75–2.67)	0.339
Age (≥60 vs. <60 years)	0.47 (0.21–1.05)	0.065	0.41 (0.16–1.05)	0.064	0.89 (0.51–1.58)	0.698	0.70 (0.37–1.34)	0.286
Gender (male vs. female)	1.50 (0.60–3.74)	0.385	1.16 (0.44–3.05)	0.767	1.46 (0.74–2.85)	0.275	1.21 (0.58–2.53)	0.610
GC location (cardiac vs. non-cardiac)	1.89 (0.88–4.10)	0.105	1.38 (0.58–3.29)	0.470	**2.32 (1.31–4.08)**	**0.004**	**2.03 (1.07–3.85)**	**0.029**
Differentiation (poor vs. well & moderate)	0.88 (0.40–1.96)	0.754	0.75 (0.32–1.74)	0.504	1.15 (0.63–2.11)	0.649	1.29 (0.67–2.50)	0.452
Vessel embolus (yes vs. no)	1.59 (0.64–3.99)	0.319	1.03 (0.34–3.12)	0.964	1.06 (0.50–2.27)	0.880	0.81 (0.34–1.97)	0.644
TNM stage (II–III vs. I)	2.46 (0.85–7.15)	0.098	2.15 (0.68–6.81)	0.194	**3.82 (1.51–9.66)**	**0.005**	**4.07 (1.39–11.93)**	**0.011**
Chemotherapy status (yes vs. no)	1.37 (0.77–2.45)	0.284	1.07 (0.56–2.05)	0.828	1.05 (0.66–1.67)	0.845	0.85 (0.49–1.46)	0.559

Bold values indicate statistical significance *p* < 0.05

## Discussion

Metastasis is the leading cause of GC-related death. Early prediction of the metastatic potential of GC is helpful for the clinical management of patients with the disease. Because it is impossible to identify cancer metastatic potential according to histopathologic or imaging examinations, efforts have been made to characterise molecular biomarkers as a predictor of metastasis of cancers, including GC.^6–9,25^ Several studies have reported biomarkers to predict recurrence or metastasis in GC patients after surgery, including target DNA methylation and specific RNA and protein expression. Although a number of molecular changes were associated with the relapse of GCs,^8,9,26–29^ their utility has not been confirmed by independent studies, or most of those studies were retrospective studies. Recently, we reported that GFRA1m and ZNF382m were consistently associated with a low risk of GC metastasis in Chinese, Japan, and Korea patients.^11^ Quantitative MethyLight assays have been established and validated for detection of the methylation level for these genes.^11,20–23^ In this prospective study, we further validated that patients with ZNF382m-high or GFRA1m-high pN_0_M_0_-GC exhibited a significantly low metastasis risk compared with those with ZNF382m-low or GFRA1m-low GC. The combination of ZNF382m-low and GFRA1m-low also had a synergistic effect on predicting GC metastasis. These data were further confirmed by the internal cross-validation analysis and mining results using TCGA methylome datasets.

*ZNF382* is a proapoptotic tumour suppressor that represses oncogenes in numerous cancers.^30^ It was hypermethylated in gastric cancer, and methylation of *ZNF382* exhibited a low risk of relapse and favourable outcomes despite their reported tumour-suppressive functions.^11^ GDNF family receptor a 1 (GFRA1) has been implicated in the regulation of neuronal cell survival and differentiation and has a role in the progression and metastasis of human cancers, such as breast cancer and pancreatic cancer, in that it promotes migration and invasion.^31–33^ Through large-scale screening and characterisation of approximately 100 genes, we found that *GFRA1* and *ZNF382* were two of 15 genes whose promoter DNA methylation status was significantly changed in GCs compared with SMs and normal/gastritis biopsies from noncancer patients.^11^ In addition, *GFRA1* and *ZNF382* methylation changes were consistently associated with GC metastasis in Chinese discovery and testing cohorts and Japanese and Korean validation cohorts. The results of the prospective study confirmed the reported findings and revealed a strong application potential. These results demonstrate that *GFRA1* and *ZNF382* methylation-related GCs may be one type of cancer with low metastasis risk. Further studies assessing whether GFRA1m and ZNF382m associate with low metastatic risk of other cancers are warranted.

In the present study, we also analysed *SRF* methylation, which was concluded as a biomarker candidate for the prediction of GC metastasis based on our previous study.^11^ Because *SRF* methylation was detected in only 19 of 178 (10.6%) SM and 13 (7.3%) GC tissue samples. The number of *SRF* methylation-positive samples was too small for the analysis of the association of *SRF* methylation with clinical characteristics. Therefore, we did not analyse *SRF* methylation data.

In this prospective study, we observed that ZNF382m and GFRA1m were significant independent predictors of metastasis for patients with pN_0_M_0_ GC. Nevertheless, there were several limitations to the study. First, the sensitivity and specificity of the combined predictive biomarker was only 61.5% and 70.1%, respectively. Further studies should be performed to optimise the current methylation marker panel to improve the predictive sensitivity and specificity. Second, the present study was based on only one centre. Thus, it is worth to further validate the predictive efficacy of these methylation markers in a multi-institutional prospective study.

## Conclusion

Our study indicates that GFRA1m and ZNF382m are potential biomarkers for the prediction of pN_0_M_0_ GC metastasis. The combination of GFRA1m and ZNF382m has a synergistic effect on predicting GC metastasis and could substantially contribute to improved management of gastric cancer patients in the context of perioperative chemotherapy. A multicentre prospective study to validate the application value of this gene methylation panel as a GC metastasis predictor is warranted.

## Supplementary information

Supp. Figure legends and Supp. Tables

## Data Availability

Requests for data and reagents can be made by contacting the corresponding or senior authors.
