# Supplementary material for: A panel of DNA methylated markers predicts metastasis of pN0M0 gastric carcinoma: a prospective cohort study
Source: Br J Cancer. 2019 Aug 21;121(7):529–36. doi: 10.1038/s41416-019-0552-0 (PMC6889426; doi:10.1038/s41416-019-0552-0)
Supplement: Supplementary file 1 — Supp. Figure legends and Supp. Tables [file 41416_2019_552_MOESM1_ESM.doc]

**Supplementary Figure and Legends**

**
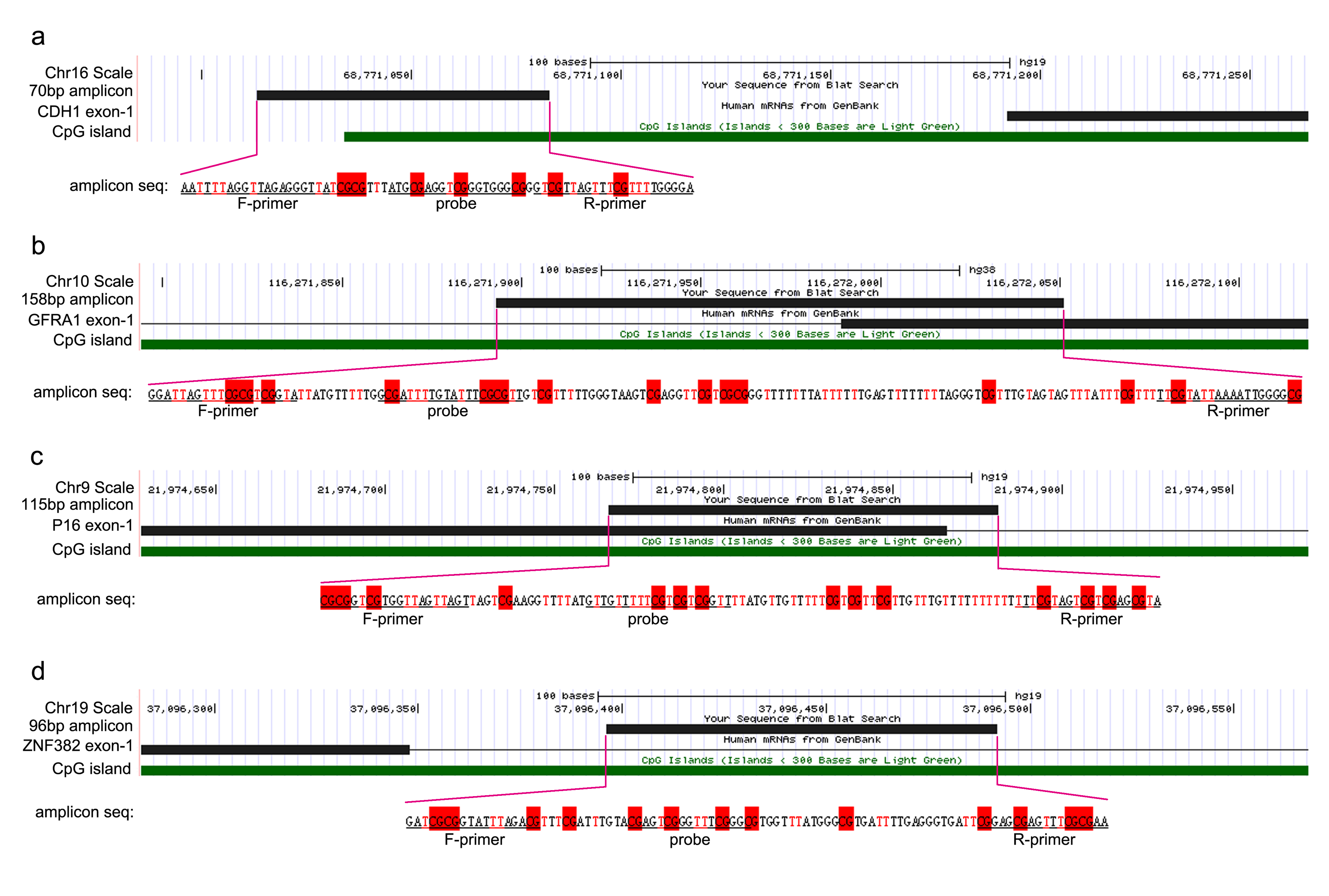
**

**Supp. Fig. 1** Locations of amplicons, primers, and probes used in MethyLight assays. **a**, *CDH1*; **b**, *GFRA1*; **c**, *P16*; **d**, *ZNF382*; red highlighted, CpG site; red letter “T”, bisulfite-converted cytosine

**
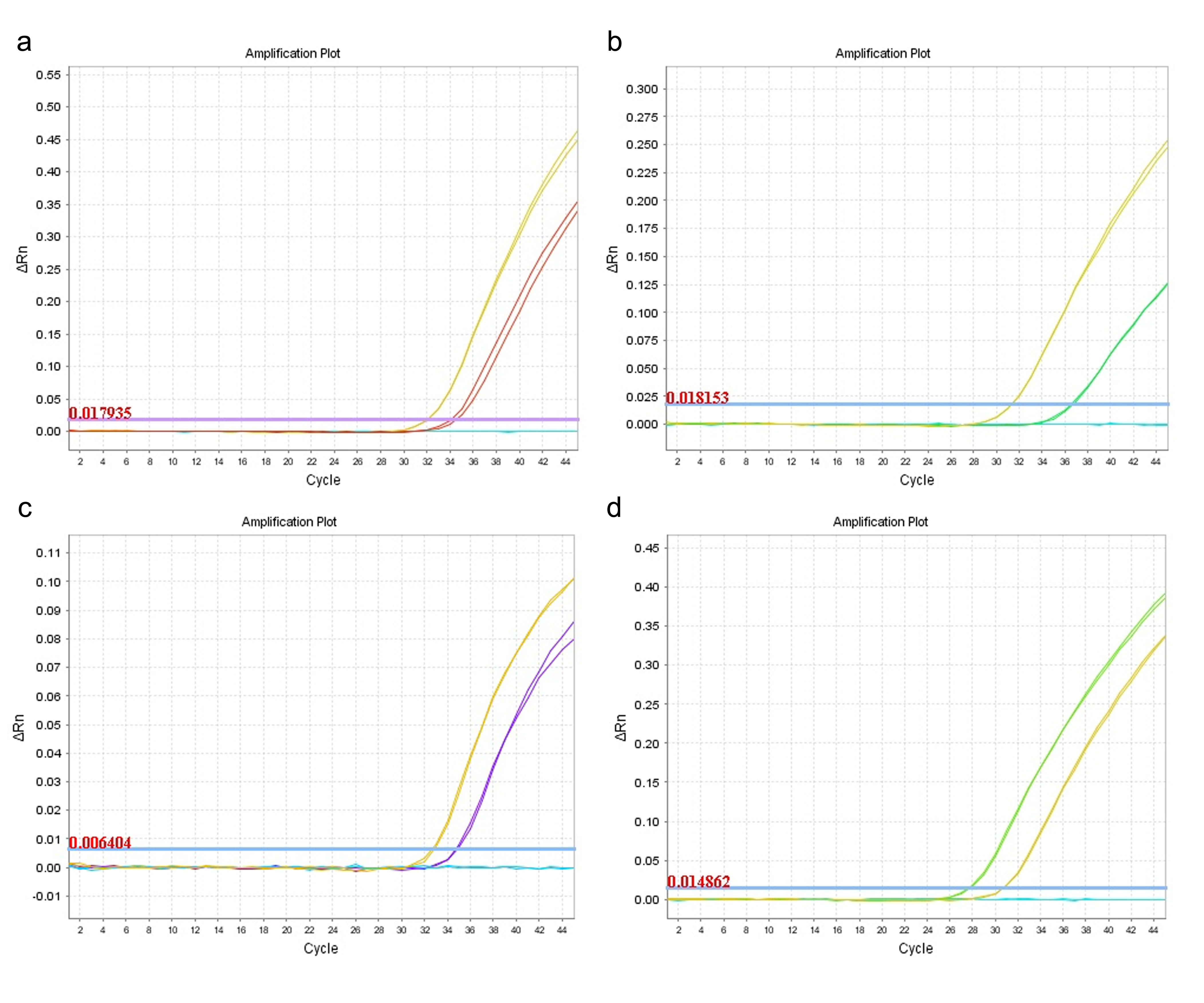
**

**Supp. Fig. 2** Amplification curves for representative samples with the MethyLight PCR.
**a**, *CDH1*; **b,**. *GFRA1*; **c**, *P16*; **d**, *ZNF382*

**
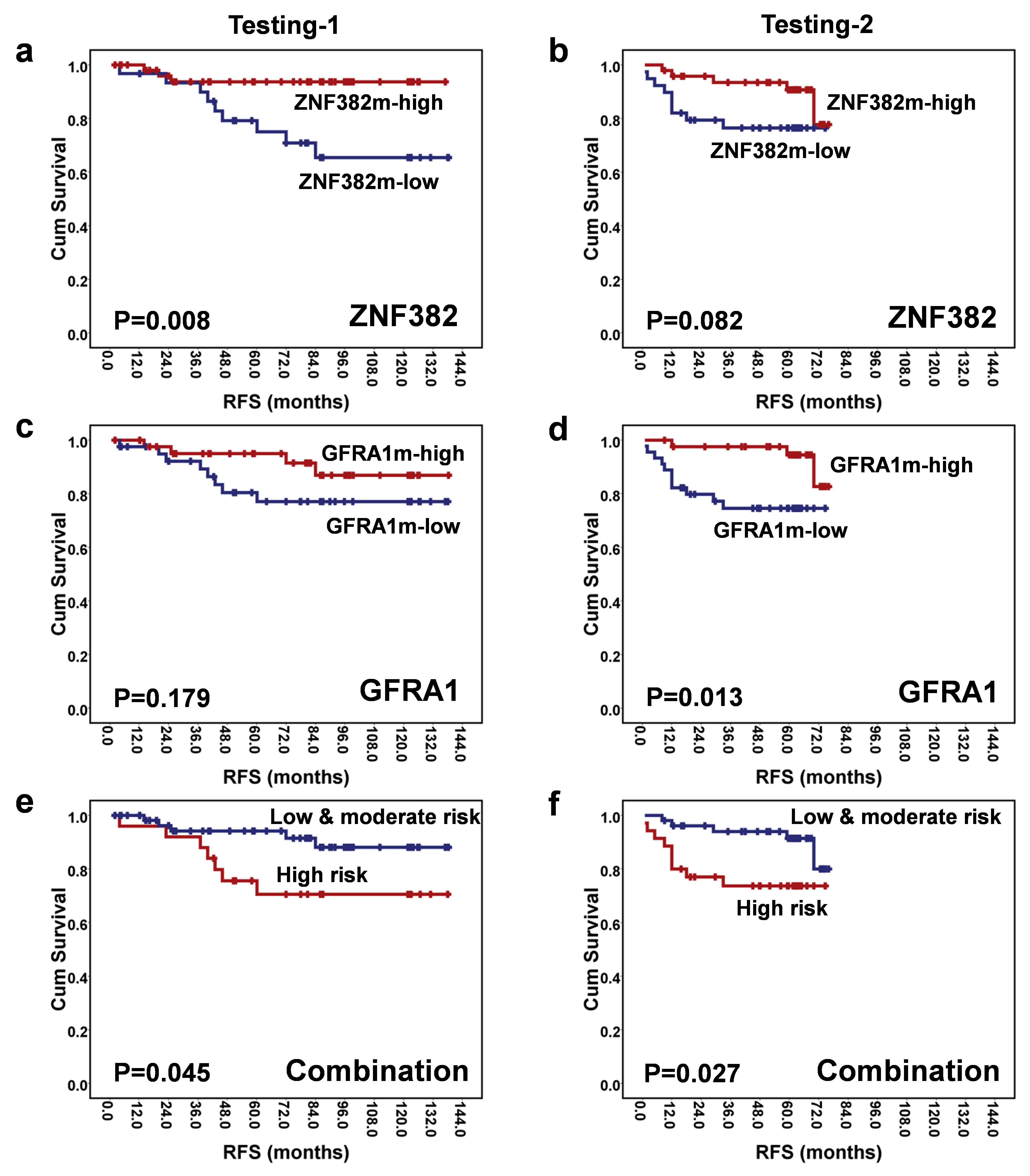
**

**Supp. Fig. 3** Kaplan-Meier survival curves of gastric carcinoma (GC) patients with different GFRA1/ZNF382 methylation statuses and numbers of risk factors in GC tissues. In testing-1 set, **a**, patients with ZNF382m-high GC had a significantly longer RFS than those with ZNF382m-low GC (adjusted HR: 0.24, 95% CI 0.06-0.96); **c**, the RFS of patients with GFRA1m-high GC was also longer compared with those with GFRA1m-low GC, but not significant; **e**, patients in the high-risk group had a shorter RFS than those in the low- and moderate-risk groups (HR: 3.06, 95% CI 1.00-9.65). In the testing-2 set, **b**, patients with ZNF382m-high GC had a longer RFS than those with ZNF382m-low GC, although not significant; **d**, the RFS of patients with GFRA1m-high GC was also longer compared with those with GFRA1m-low GC (HR: 0.18, 95% CI 0.04-0.87). **f**, Patients in the high-risk group had a shorter RFS than those in the low- and moderate-risk groups (HR: 3.23, 95% CI 1.07-9.72)

**
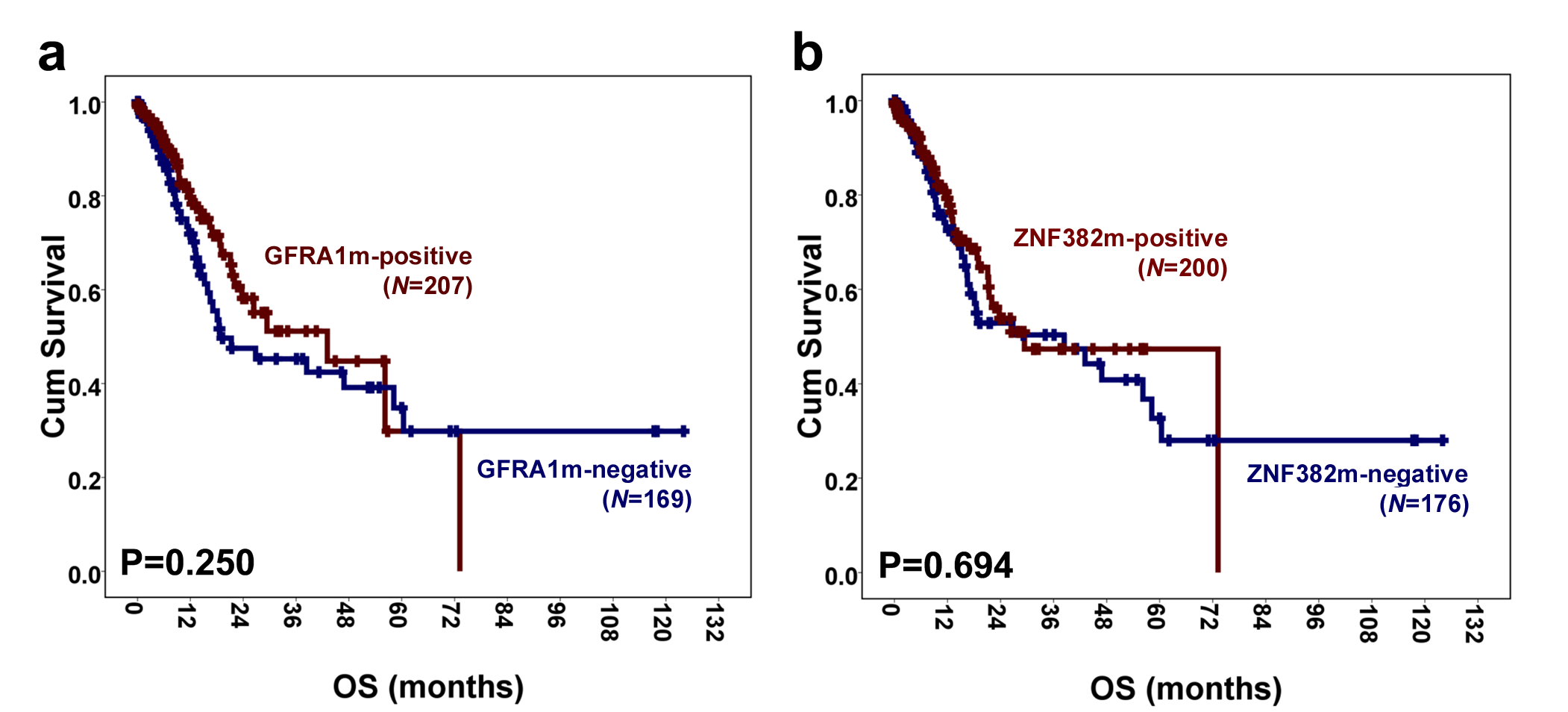
**

**Supp. Fig. 4** Kaplan-Meier OS curves of GC patients from TCGA datasets with different *GFRA1*
and *ZNF382* methylation statuses. **a**, *GFRA1*; **b**, *ZNF382*.

**Supplementary Tables**

**Supp. Table 1. Gene-specific primer and probe sequences for MethyLight and quantitative RT-PCR assays**

| Gene | Primer and probe oligo DNA sequences (5’-3’) | Amplicon  size (bp) | PCR annealing temperature (°C) |
| --- | --- | --- | --- |
| *CDH1* | Forward: aattttaggttagagggttatcgcg Reverse: tccccaaaacgaaactaacgac Probe: 6FAM-cgcccacccgacctcgcat -TAMRA | 70 | 56.0 |
| *GFRA1* | Forward: ggattagtttcgcgtcggta Reverse: cgccccaattttaatacgaa Probe: 6FAM- aacgcgaaatacaaaatcgc-TAMRA | 158 | 58.5 |
| *P16* | Forward: cgcggtcgtggttagttagt Reverse: tacgctcgacgactacgaaa Probe: 6FAM- gttgtttttcgtcgtcggtt-TAMRA | 115 | 62.0 |
| *ZNF382* | Forward: gatcgcggtatttagacg Reverse: ttcgcgaaactcgctccg Probe: 6FAM-gcccgaaacccgactcg-TAMRA | 96 | 58.0 |
| *SRF* | Forward: gggagtgtcgggttgagtc | 116 | 56.0 |
|  | Reverse: ccccaacttaaatcaataacaaaca |  |  |
|  | Probe: 6FAM-aagtcgatggcggcggtt-TAMRA |  |  |
| *COL2A1* | Forward: tctaacaattataaactccaaccaccaa Reverse: gggaagatgggatagaagggaatat Probe:6FAM-ccttcattctaacccaatacctatcccacctctaaa-TAMRA | 91 | 56.0 |

**Supp. Table 2.** Comparison of the prevalence of gastric carcinoma (GC) metastasis in patients with different levels of methylated *ZNF382* CpG islands (ZNF382m) in GC tissue samples

|  |  | ZNF382m proportion, *median* (25%-75% *range*) (%) | Metastasis rate (%) | | | OR (95% CI), ZNF382m-high versus ZNF382m-low | |
| --- | --- | --- | --- | --- | --- | --- | --- |
|  |  |  | (All patients) | Patients with ZNF382m-high GC ^a^ | Patients with ZNF382m-low GC | Univariate analysis | Multivariate analysis |
| Sex | Male | 10.0 (2.7-21.1) | 20/121 (16.5) | 6/70 (8.6) | 14/51 (27.5) | 0.25 (0.09-0.71) | 0.17 (0.05-0.55) |
|  | Female | 12.4 (3.4-22.2) | 6/52 (11.5) | 2/33 (6.1) | 4/19 (21.1) |  |  |
| Age (yrs) | <60 | 8.4 (2.1-20.8) | 17/83 (20.5) | 3/44 (6.8) | 14/39 (35.9) | 0.13 (0.04-0.51) | 0.09 (0.02-0.42) |
|  | ≥60 | 12.5 (4.0-23.0) | 9/90 (10.0) | 5/59 (8.5) | 4/31 (12.9) |  |  |
| Location | Noncardiac | 12.6 (4.1-22.8) ^b^ | 14/115 (12.2) | 5/75 (6.7) | 9/40 (22.5) | 0.25 (0.08-0.81) | 0.20 (0.06-0.69) |
|  | Cardiac | 6.6 (1.5-17.0) | 12/58 (20.7) | 3/28 (10.7) | 9/30 (30.0) |  |  |
| Different | Well/mod | 13.3 (3.0-24.5) | 10/62 (16.1) | 2/37 (5.4) | 8/25 (32.0) | 0.13 (0.02-0.65) | 0.09 (0.01-0.68) |
|  | Poor | 10.7 (2.9-20.9) | 15/105 (14.3) | 6/64 (9.4) | 9/41 (22.0) |  |  |
| Vessel | Negative | 11.7 (2.8-22.2) | 19/143 (13.3) | 5/86 (5.8) | 14/57 (24.6) | 0.19 (0.07-0.57) | 0.16 (0.05-0.49) |
| embolus | Positive | 8.6 (3.4-20.8) | 6/27 (22.2) | 2/16 (12.5) | 4/11 (36.4) |  |  |
| TNM  stage | I | 8.3 (1.6-20.9) | 4/51 (7.8) | 0/28 (0.0) | 4/23 (17.4) |  |  |
|  | II | 12.0 (2.9-22.8) | 20/100 (20.0) | 8/62 (12.9) | 12/38 (31.6) |  |  |
|  | III | 11.0 (4.5-16.2) | 2/22 (9.1) | 0/13 (0.0) | 2/9 (22.2) |  |  |
| Chemotherapy | No | 10.0 (3.5-19.6) | 10/97 (10.3) | 2/57 (3.5) | 8/40 (20.0) | 0.15 (0.03-0.73) | 0.15 (0.03-0.79) |
|  | Yes | 11.9 (2.5-22.6) | 15/73 (20.5) | 5/44 (11.4) | 10/29 (34.5) | 0.25 (0.08-0.84) | 0.18 (0.05-0.67) |
| (Total) |  | 10.5 (2.8-21.1) | 26/173 (15.0) | 10/103 (9.7) | 16/70 (22.9) | 0.25 (0.10-0.60) | 0.17 (0.06–0.47) |

^a^ Cutoff value for ZNF382m-high in GC tissue samples, >6.84%. ^b^, Mann–Whitney U-test, *P*=0.025.

**Supp. Table 3.** Comparison of the prevalence of gastric carcinoma (GC) metastasis/recurrence in patients with
different levels of methylated *GFRA1* CpG islands (GFRA1m) in GC tissue samples

|  |  | GFRA1m proportion, *median* (25%-75% *range*) (%) | Metastasis rate (%) | | OR (95% CI), GFRA1m-high versus GFRA1m-low | |
| --- | --- | --- | --- | --- | --- | --- |
|  |  |  | Patients with GFRA1m-high GC ^a^ | Patients with GFRA1m-low GC | Univariate analysis | Multivariate analysis |
| Sex | Male | 6.4 (0.9-21.3) | 4/56 (7.1) | 16/65 (24.6) | 0.23 (0.07-0.74) | 0.11 (0.02-0.53) |
|  | Female | 12.4 (2.4-26.2) | 3/30 (10.0) | 3/22 (13.6) |  |  |
| Age (yrs) | <60 | 7.5(1.2-18.8) | 2/40 (5.0) | 15/43 (34.9) | 0.01 (0.02-0.45) | 0.05 (0.01-0.37) |
|  | ≥60 | 10.8 (0.9-26.7) | 5/46 (10.9) | 4/44 (9.1) |  |  |
| Location | Noncardiac | 10.6(1.7-25.4) ^b^ | 5/64 (7.8) | 9/51 (17.6) |  |  |
|  | Cardiac | 3.3 (0.3-19.2) | 2/22 (9.1) | 10/36 (27.8) |  |  |
| Different. | Well/mod | 6.7 (0.5-21.9) | 1/29 (3.4) | 9/33 (27.3) | 0.09 (0.01-0.77) |  |
|  | Poor | 9.4 (1.9-25.2) | 6/55 (10.9) | 9/50 (18.0) |  |  |
| Vessel | Negative | 8.6 (1.0-24.5) | 4/71 (5.6) | 15/72 (20.8) | 0.22 (0.07-0.71) | 0.23 (0.07-0.75) |
| embolus | Positive | 9.4(1.6-21.1) | 2/14 (14.3) | 4/13 (30.8) |  |  |
| TNM  stage | I | 6.1 (0.9-14.9) | 1/25 (4.0) | 3/26 (11.5) |  |  |
|  | II | 10.1 (1.7-26.4) | 5/51 (9.8) | 15/49 (30.6) | 0.26 (0.09-0.80) | 0.15 (0.04-0.58) |
|  | III | 3.7 (0.7-23.5) | 1/10 (10.0) | 1/12 (8.3) |  |  |
| Chemotherapy | No | 8.7 (1.4-19.2) | 2/49 (4.1) | 8/48 (16.7) |  |  |
|  | Yes | 7.3 (0.9-26.2) | 4/34 (11.8) | 11/39 (28.2) |  |  |
| (Total) |  | 8.6 (1.1-23.6) | 7/86 (8.1) | 19/87 (21.8) | 0.31 (0.12-0.79) | 0.23 (0.08-0.66) |

^a^ Cutoff value for GFRA1m-high in GC samples, >8.64%. ^b^, Mann–Whitney U-test: *P*=0.026.

**Supp. Table 4.** Prevalence of gastric carcinoma (GC) metastasis during follow-up in patients in different risk groups

|  | | Testing-set 1 | | |  | Testing-set 2 | | |
| --- | --- | --- | --- | --- | --- | --- | --- | --- |
|  |  | GC metastasis rate (%) | | Univariate analysis |  | GC metastasis rate (%) | | Univariate analysis |
|  |  | Patients in the low & mod. risk groups | Patients in the high risk group | OR (95% CI), high vs. low & mod. risk groups |  | Patients in the low & mod. risk groups | Patients in the high risk group | OR (95% CI), high vs. low & mod. risk groups |
| Sex | Male | 3/38 (7.9) | 6/20 (30.0) | **5.00 (1.10-22.82)^a^** |  | 4/37 (10.8) | 7/26 (26.9) | 3.04 (0.79-11.75) |
|  | Female | 2/23 (8.7) | 1/5 (20.0) | 2.63 (0.19-36.34) |  | 1/15 (6.7) | 2/9 (22.2) | 4.00 (0.31-52.07) |
| Age (yrs) | <60 | 3/30 (10.0) | 5/15 (33.3) | 4.50 (0.90-22.40) |  | 1/19 (5.3) | 8/19 (27.6) | **13.09 (1.44-119.34)^b^** |
|  | ≥60 | 2/31 (6.5) | 2/10 (20.0) | 3.63 (0.44-29.91) |  | 4/33 (12.1) | 1/16 (6.3) | 0.48 (0.05-4.72) |
| Location | Noncardiac | 3/41 (7.3) | 2/13 (15.4) | 2.30 (0.34-15.57) |  | 3/41 (7.3) | 6/20 (30.0) | **5.43 (1.19-24.71)** |
|  | Cardiac | 2/20 (10.0) | 5/12 (41.7) | **6.43 (1.00-41.20)** |  | 2/11 (18.2) | 3/15 (20.0) | 1.13 (0.15-8.21) |
| Differentiation | Well/ Mod. | 2/23 (8.7) | 3/7 (42.9) | 7.88 (0.98- 63.31) |  | 1/19 (5.3) | 4/13 (30.8) | 8.00 (0.78- 82.46) |
|  | Poor | 3/37 (8.1) | 3/15 (20.0) | 2.83 (0.52- 15.99) |  | 4/32 (12.5) | 5/21 (23.8) | 2.19 (0.51-9.34) |
| Vessel  embolus | Negative | 4/52 (7.7) | 3/17 (17.6) | 2.57 (0.51- 12.88) |  | 3/42 (7.1) | 9/32 (28.1) | **5.09 (1.25- 20.72)^c^** |
|  | Positive | 0/8 (0.0) | 4/6 (66.7) | 0.00 (0.00- ) |  | 2/10 (20.0) | 0/3 (0.0) | 0.00 (0.00-) |
| pTNM  Stage | I | 1/12 (8.3) | 0/6 (0.0) | 0.00 (0.00- ) |  | 0/22 (0.0) | 3/11 (27.3) | 0.00 (0.00-) |
|  | II | 3/37 (8.1) | 6/14 (42.9) | **8.50 (1.74- 41.50)** |  | 5/28 (17.9) | 5/21 (23.8) | 1.44 (0.36- 5.80) |
|  | III | 1/12 (8.3) | 1/5 (20.0) | 2.75 (0.14- 55.17) |  | 0/2 (0.0) | 1/3 (33.3) | 0.00 (0.00-) |
| Chemotherapy | No | 1/27 (3.7) | 3/13 (23.1) | 7.80 (0.72-84.) |  | 2/37 (5.4) | 4/20 (20.0) | 4.67 (0.77-28.29) |
|  | Yes | 4/32 (12.5) | 4/12 (33.3) | 3.50 (0.71-17.22) |  | 2/14 (14.3) | 5/15 (33.3) | 4.00 (0.54-29.81) |
| (Total) |  | 5/61 (8.2) | 7/25 (28.0) | **4.36 (1.23-15.43)^d^** |  | 5/52 (9.6) | 9/35 (25.7) | **3.25 (1.00- 10.73)** |

^a/b/c/d^, multivariate analysis, OR (95% CI), 13.81 (1.42- 134.22)/56.67 (2.18-147.16)/4.71 (1.03-21.55)/5.48 (1.19-25.31).

**Supp. Table 5.** Comparison of ZNF382m- and GFRA1m-high/positive rates in gastric carcinoma(GC) patients with various clinicopathological characteristics in the prospective cohort and TCGA database

| Clinicopathological features |  | Patients (*N*=173) in the prospective cohort | |  | Patients (*N*=376)in TCGA database | |
| --- | --- | --- | --- | --- | --- | --- |
|  |  | GFRA1m-high^a^ rate (%) | ZNF382m-high^b^ rate (%) |  | GFRA1m-positive rate (%) | ZNF382m-positive rate (%) |
| Sex | Male | 57/121 (47.1) | 70/121 (57.9) |  | 147/244 (60.2) | 140/244(57.4) |
|  | Female | 29/52 (55.8) | 33/52 (63.5) |  | 69/132(52.3) | 66/132 (50.0) |
| Age (yrs) | <60 | 39/82 (47.6) | 43/82 (52.4) |  | 61/120(50.8) | 57/120(47.5) |
|  | ≥60 | 47/91 (51.6) | 60/91 (65.9) |  | 151/252(60.0) | 146/252(57.9) |
| Location | Noncardiac | 64/115 (55.7)^c^ | 75/115 (65.2)^d^ |  | 163/275(59.3) | 150/275(54.5) |
|  | Cardiac | 22/58 (37.9) | 28/58 (48.3) |  | 52/94(55.3) | 54/94(57.4) |
| Differentiation | Well/mod | 30/62 (48.4) | 37/62 (59.7) |  | 114/175 (65.1)^e^ | 110/175 (62.9)^f^ |
|  | Poor | 54/105 (51.4) | 64/105 (61.0) |  | 34/75 (45.3) | 29/75 (38.7) |
| Vascular | Negative | 71/143 (49.7) | 86/143 (60.1) |  | NA | NA |
| embolism | Positive | 14/27 (51.9) | 16/27 (59.3) |  | NA | NA |
| Local invasion | T_1-2_ | 24/51 (47.1) | 28/51 (54.9) |  | 50/85(58.8) | 49/85(57.6) |
|  | T_3_ | 51/99 (51.5) | 61/99 (61.6) |  | 81/138(58.7) | 77/138(55.8) |
|  | T_4_ | 11/23 (47.8) | 14/23 (60.9) |  | 85/153(55.6) | 80/153(52.3) |
| Lymph metastasis | N_0_ | 86/173 (49.7) | 103/173 (59.5) |  | 68/119(57.1) | 66/119(55.5) |
|  | N_1_ | 0 | 0 |  | 47/76(61.8) | 45/76(59.2) |
|  | N_2_ | 0 | 0 |  | 47/77(61.0) | 40/77(51.9) |
|  | N_3_ | 0 | 0 |  | 50/99(50.5) | 50/99(50.5) |
| Distant metastasis | M_0_ | 86/173 (49.7) | 103/173 (59.5) |  | 207/353(58.6) | 198/353(56.1)^g^ |
|  | M_1_ | 0 | 0 |  | 9/23(39.1) | 8/23 (34.8) |
| Chemotherapy | No | 35/78 (44.9) | 41/78 (52.6) |  |  |  |
|  | Yes | 30/65 (46.2) | 39/65 (60.0) |  | 216/376 (57.4) | 206/276 (74.6) |
| Race | Asian | 86/173 (49.7) | 103/173 (59.5) |  | 63/87 (72.4)^h^ | 57/87 (65.5)^i^ |
|  | Caucasian | 0 | 0 |  | 121/238 (50.8) | 120/238 (50.4) |
|  | Black | 0 | 0 |  | 6/11 (54.5) | 6/11 (54.5) |

^a^ Cutoff value for GFRA1m-high GC tissue samples, >8.64%. ^b^ Cutoff value for ZNF382m-high GC tissue samples, >6.84%. ^c/d/e/f/g^, Chi-square test, P=0.028/0.032/0.003/＜0.001/ 0.047; ^h/i^, Chi-square test, Asian vs.Caucasian, P=0.001/0.016.

**Supp. Table 6.** Univariate and multivariate analysis of the association between *GFRA1* methylation (GFRA1m)
or *ZNF382* methylation (ZNF382m) and relapse-free survival (RFS) of gastric carcinoma (GC) patients

| Clinical parameter | Univariate analysis | |  | Multivariate analysis | |
| --- | --- | --- | --- | --- | --- |
|  | HR (95% CI) | *P-*value |  | HR (95% CI) | *P-*value |
| GC-ZNF382m (high vs. low) | **0.28 (0.12-0.66)** | **0.003** |  | **0.24 (0.09-0.61)** | **0.003** |
| GC-GFRA1m (high vs. low) | **0.32 (0.13-0.76)** | **0.010** |  | **0.26 (0.10-0.72)** | **0.009** |
| Age (≥60 vs. <60 years) | 0.47 (0.21-1.05) | 0.065 |  | 0.45 (0.17-1.20) | 0.110 |
| Gender (male vs. female) | 1.50 (0.60-3.74) | 0.385 |  | 1.31 (0.50-3.45) | 0.578 |
| GC location (cardiac vs. noncardiac) | 1.89 (0.88-4.10) | 0.105 |  | 1.47 (0.58-3.69) | 0.418 |
| Differentiation (poor vs. well & moderate) | 0.88 (0.40-1.96) | 0.754 |  | 0.75 (0.32-1.74) | 0.499 |
| Vessel embolus (yes vs. no) | 1.59 (0.64-3.99) | 0.319 |  | 0.88 (0.29-2.72) | 0.826 |
| TNM stage | 1.31 (0.73-2.34) | 0.363 |  | 1.15 (0.54-2.43) | 0.724 |
| Chemotherapy status (yes vs. no) | 1.93 (0.87-4.32) | 0.107 |  | 1.76 (0.70-4.39) | 0.229 |
